# Differential effects of cyclophosphamide and mycophenolate mofetil on cellular and serological parameters in patients with systemic lupus erythematosus

**DOI:** 10.1186/s13075-015-0603-8

**Published:** 2015-04-03

**Authors:** Till Fassbinder, Ute Saunders, Eva Mickholz, Elisabeth Jung, Heidemarie Becker, Bernhard Schlüter, Annett Marita Jacobi

**Affiliations:** Division of Rheumatology and Clinical Immunology/ Department of Internal Medicine D, University Hospital Münster, Albert-Schweitzer-Campus 1, Building A1, 48149 Münster, Germany; Division of Rheumatology and Clinical Immunology, Brandenburg Medical School, Fehrbelliner Str. 38, 16816 Neuruppin, Germany; Center for Laboratory Medicine, University Hospital Münster, Albert-Schweitzer-Campus 1, Building A1, 48149 Münster, Germany

## Abstract

**Introduction:**

Disease activity and therapy show an impact on cellular and serological parameters in patients with systemic lupus erythematosus (SLE). This study was performed to compare the influence of mycophenolate mofetil (MMF) and cyclophosphamide (CYC) therapy on these parameters in patients with flaring, organ-threatening disease.

**Methods:**

SLE patients currently receiving CYC (n = 20), MMF (n = 25) or no immunosuppressive drugs (n = 22) were compared using a cross-sectional design. Median disease activity and daily corticosteroid dose were similar in these treatment groups. Concurrent medication, organ manifestations, and disease activity were recorded, and cellular and serological parameters were determined by routine diagnostic tests or flow cytometric analysis. In addition follow-up data were obtained from different sets of patients (CYC n = 24; MMF n = 23).

**Results:**

Although both drugs showed a significant effect on disease activity and circulating B cell subsets, only MMF reduced circulating plasmablasts and plasma cells as well as circulating free light chains within three months of induction therapy. Neither MMF nor CYC were able to reduce circulating memory B cells. MMF lowered IgA levels more markedly than CYC. We did not observe a significant difference in the reduction of IgG levels or anti-dsDNA antibodies comparing patients receiving MMF or CYC. In contrast to MMF, induction therapy with CYC was associated with a significant increase of circulating CD8+ effector T cells and plasmacytoid dendritic cells (PDCs) after three months.

**Conclusions:**

The results indicate differences between MMF and CYC with regard to the mechanism of action. MMF, but not CYC, treatment leads to a fast and enduring reduction of surrogate markers of B cell activation, such as circulating plasmablasts, plasma cells and free light chains but a comparable rate of hypogammaglobulinemia.

**Electronic supplementary material:**

The online version of this article (doi:10.1186/s13075-015-0603-8) contains supplementary material, which is available to authorized users.

## Introduction

Systemic lupus erythematosus (SLE) is a chronic autoimmune disease associated with significant morbidity and mortality. SLE is a heterogeneous disease involving hematologic, neurologic, dermatologic, musculoskeletal and renal organ systems. Primarily young women are affected by SLE (female:male ratio of 6:1 to 10:1) requiring treatment with immunosuppressive drugs and other medications [1,2]. Treatment guidelines based on clinical trials have been published recently [3-5]. However, due to the heterogeneity of the disease, employment of immunosuppressive drugs is largely based on clinical experience [6]. Besides hydroxychloroquine and prednisone, antiproliferative or cytotoxic reagents, such as azathioprine (AZA), methotrexate (MTX), mycophenolate mofetil (MMF) or cyclophosphamide (CYC), are used to treat SLE. These drugs are able to reduce morbidity and mortality, while discontinuation often results in a relapse of the disease.

The pathogenesis of SLE is complex. A loss of tolerance to self-antigens as well as a dysregulated T and B cell activation are implicated in the pathogenesis of SLE [7]. In particular, activation of B cells and the loss of B cell tolerance play a pivotal role in SLE, because B cells present antigens, produce numerous autoantibodies and proinflammatory cytokines and activate T cells [8]. In this context, alterations of circulating lymphocyte and dendritic cell subsets have been observed, such as plasma cells [9], transitional B cells [10], pre-switched memory B cells [11,12], regulatory T cells [13], CD4^−^CD8^−^ T cells [14] or plasmacytoid dendritic cells (PDC) [15]. However, it is not clear, if these abnormalities are related to disease activity, therapeutic interventions, or both. By comparing patients receiving maintenance therapy with AZA or MMF using a cross-sectional design, we observed that different drugs used for the same purpose target distinct cell subsets, as for instance MMF blocks plasma cell differentiation whereas AZA reduces naïve and transitional B cells [16,17]. However, our knowledge about the mechanism of action of many drugs used to treat lupus is still limited. Therefore, we continued to investigate the effects of immunosuppressive drugs used to induce or maintain remission in patients with SLE.

## Methods

### Patients

All data were obtained from patients fulfilling the American College of Rheumatology (ACR) criteria for the classification of SLE [18,19] attending the Division of Rheumatology and Clinical Immunology of the Department of Internal Medicine D at Münster University Hospital. Patients gave written informed consent to a retrospective analysis of all data acquired during their routine visits. Ethical approval for retrospective analysis of serological, clinical and cellular data obtained to assess disease activity or safety of treatment was waived by the ‘Ethik-Kommission der Ärztekammer Westfalen-Lippe und der Medizinischen Fakultät der Westfälischen Wilhelms Universität Münster’. Patients included in the analysis had to be on their medication for at least ten weeks.

For a cross-sectional analysis, cellular, serological, and clinical parameters were recorded in patients receiving immunosuppressive therapy with MMF (n = 25) or CYC (n = 20). The following primary features, that is, nephritis (n = 18), mucocutaneous (n = 3); arthritis (n = 3) or myositis (n = 1) in the MMF group and nephritis (n = 17), myositis (n = 2) or alveolitis (n = 1) in the CYC group led to the respective therapy. In addition, all manifestations still active at the time point of analysis are given in Table 1. Since it is known that corticosteroid intake and disease activity influence cellular and serological parameters, patient groups showing similar median disease activity or daily corticosteroid dose were compared as shown in Table 1. Patients treated with MMF had been receiving a daily dose of 1,000 mg (n = 2), 1,500 mg (n = 7), 2,000 mg (n = 12), 2,500 mg (n = 1) or 3,000 mg (n = 3) MMF for ten weeks to six month (n = 4), six to twelve months (n = 2) or more than one year (n = 19) prior to analysis. All patients in the CYC group received intravenous (i.v.) pulses. Of these, the majority (n = 15) was analyzed after CYC treatment according to the Euro-Lupus Trial protocol [20]. They received 0.5 g CYC every two weeks for a total of twelve weeks. The remaining five patients received i.v. CYC once every four weeks for four to twelve months. For comparison another group of lupus patients (n = 22) with similar disease activity and daily corticosteroid use was identified and parameters of these patients were recorded prior to increasing treatment intensity (controls). The clinical and demographic data as well as medication and serological parameters of these patients are shown in Table 1.

**Table 1:** **Demographic, serological, clinical data and medication of patients treated with MMF or CYC compared to controls**

**Demographic, serological, clinical data and medication**	**MMF**	**CYC**	**Controls**
	**(number = 25)**	**(number = 20)**	**(number = 22)**
**SLEDAI-2 k** (points); median (range)	8 (2 to 14)	11 (0 to 16)	9 (3 to 18)
**gender** female number (%)	18 (72.0)	14 (70.0)	18 (81.8)
**age** (years); mean ± SD	38 ± 13	34 ± 9	40 ± 14
**duration** (years); median (range)	9 (2 to 28)^a^	4 (0 to 25)^a^	10 (0 to 26)
**age** at initial diagnosis of SLE (years); mean ± SD	27 ± 11	29 ± 10	29 ± 14
**medication**			
prednisone (mg/day); median (range)	7.0 (2.5 to 15.0)	10.0 (0.0 to 30.0)	6.3 (0.0 to 100.0)
co-medication with antimalarials number (%)	18 (72.0)	12 (60.0)	16 (72.7)
**currently active manifestations number (%)**			
class III-V nephritis	10 (40.0)	12 (60.0)	6 (27.3)
eGFR <60 ml/min	7 (28.0)	4 (20.0)	3 (13.6)
C3c <0.9 g/L	18 (72.0)	14 (70.0)	16 (72.7)
neuropsychiatric	0	0	0
mucocutaneous/cutaneous	8 (32.0)	8 (40.0)	7 (31.8)
arthritis	5 (20.0)	0^bb^	9 (40.9)^bb^
serositis	1 (4.0)	0	4 (18.2)
myositis	1 (4.0)	1 (5.0)	1 (4.5)
**autoantibodies number (%)**			
anti-dsDNA >7 U/ml	21 (84.0)	15 (75.0)	20 (90.9)
anti-Ro >7 U/ml	11 (44.0)	10 (50.0)	13 (59.1)
anti-La >7 U/ml	4 (16.0)	4 (20.0)	4 (18.2)
anti-U1-RNP >5 U/ml	11 (44.0)	12 (60.0)	8 (36.4)
anti-SM >5 U/ml	7 (28.0)	9 (45.0)	4 (18.2)

Statistically significant differences (Dunn’s multiple comparisons test or Fisher’s exact test) were observed between patients receiving MMF and CYC (^a^) and patients receiving CYC versus controls (^b^) one (*P* <0.05), two (*P* <0.01) symbols ^ab^. C3c: complement factor C3c, controls: patients with SLE not receiving MMF or CYC; CYC: cyclophosphamide; eGFR: estimated glomerular filtration rate; MMF: mycophenolate mofetil; SLE: systemic lupus erythematosus; SLEDAI-2 k: SLE disease activity index.

For the follow-up analysis we identified 47 flaring patients requiring treatment with CYC (n = 24) or MMF (n = 23) because of severe flares of nephritis (n = 18), myositis (n = 2), neuropsychiatric manifestations (n = 3) or alveolitis (n = 1) in the CYC treated group and nephritis (n = 17), arthritis (n = 3), myositis (n = 1), vasculitis (n = 1) or neuropsychiatric manifestations (n = 1) in the MMF treated group. In addition, all manifestations active prior to treatment are given in Table 2. All subjects were investigated prior to treatment and after 15 (range: 10 to 39) weeks of i.v. CYC application (in 17 of 24 cases according to the Euro-Lupus Trial protocol [20] or 16 (10 to 65) weeks after starting MMF. Patients taking MMF received a daily dose of 1,500 mg (n = 3), 2,000 mg (n = 16) or 3,000 mg (n = 4) MMF. Again, both treatment groups showed a similar median disease activity and daily corticosteroid dose.

**Table 2:** **Demographic, serological, clinical data and medication prior to induction therapy**

**Demographic, serological, clinical data and medication**	**MMF**	**CYC**
	**(number = 23)**	**(number = 24)**
**SLEDAI-2 k** (points); median (range)	12 (6 to 20)	14 (2 to 30)
**gender** female number (%)	15 (65.2)	16 (66.7)
**age** (years); mean ± SD	35 ± 10	34 ± 10
**duration** (years); median (range)	4 (1 to 24)	3 (0 to 25)
**age** at initial diagnosis of SLE (years); mean ± SD	29 ± 10	30 ± 10
**medication**		
prednisone (mg/day); median (range)	10.0 (5.0-30.0)	10.0 (0.0-250.0)
co-medication with antimalarials number (%)	15 (65.2)	11 (45.8)
**preceding immunosuppressive medication number (%)**		
- CYC	11 (47.8)	
- MMF		6 (25.0)
- AZA	8 (24.8)	7 (29.2)
- MTX	2 (8.7)	2 (8.3)
- CsA	1 (4.3)	1 (4.2)
- none	1 (4.3)^a^	8 (33.3)^a^
**manifestations number (%)**		
class III-V nephritis flare	17 (73.9)	18 (75.0)
eGFR <60 ml/min	4 (18.2)	6 (25.0)
C3c <0.9 g/L	14 (60.9)^b^	23 (95.8)^b^
neuropsychiatric	1 (4.3)	3 (12.5)
mucocutaneous/cutaneous	14 (60.9)	14 (58.3)
arthritis	8 (34.8)	6 (25.0)
serositis	2 (8.7)	5 (20.8)
myositis	2 (8.7)	3 (12.5)
**autoantibodies number (%)**		
anti-dsDNA >7 U/ml	21 (91.3)	21 (87.5)
anti-Ro >7 U/ml	15 (65.2)	13 (54.2)
anti-La >7 U/ml	5 (21.7)	5 (20.8)
anti-U1-RNP >5 U/ml	11 (47.8)	13 (54.2)
anti-SM >5 U/ml	8 (34.8)	11 (45.8)

Statistically significant differences between patients undergoing induction therapy with CYC and MMF (Fisher’s exact test): ^a^ *P* = 0.0226; ^b^ *P* = 0.0044. AZA: azathioprine; C3c: complement factor C3c; CsA: cyclosporine A; CYC: cyclophosphamide; eGFR: estimated glomerular filtration rate; MMF: mycophenolate mofetil; MTX: methotrexate; SLE: systemic lupus erythematosus; SLEDAI-2 k: SLE disease activity index.

Data of 20 patients were analyzed repeatedly approximately 31 (18 to 111) weeks after starting MMF treatment. Since the major percentage of the patients receiving CYC was treated according to the Euro-Lupus Trial protocol [20] no further follow-up parameters could be collected for this group. Clinical, serological, demographic data and the preceding immunosuppressive medication are given in Table 2.

Furthermore, plasmablasts, plasma cells and immunoglobulin (Ig)-, as well as free light chain levels, were determined in 186 lupus patients and data were analyzed using Spearman’s rank correlation test. The mean age of these patients was 38.2 ± 12.3 years ranging from 18 to 72 years. Of these patients, 81.7% were female, 30.0% received MMF, 21.5% AZA, 9.7% MTX, 12.4% CYC, and 28.5% no immunosuppressive medication except steroids and antimalarials. The median SLEDAI-2 k of these 186 patients was 6 (0 to 24).

### Acquisition of data

Routine laboratory analyses including differential blood counts and complement factor C3c (turbidimetry), as well as autoantibody (radioimmuno- or fluorescence-enzyme-immunoassay), Ig levels (turbidimetry), and free light chain concentrations (nephelometry) were measured in the central laboratory of the University Hospital of Münster, Germany using accredited diagnostic procedures. To be considered positive autoantibody levels had to be higher than 7 U/ml (anti-dsDNA, anti-Ro, anti-La) or higher than 5 U/ml (anti-U1-RNP and anti-Sm).

Flow cytometric analysis of peripheral blood mononuclear cells (PBMC) was performed as described previously [16]. Briefly, PBMC from 5 ml of heparinized blood were isolated by density gradient centrifugation using Ficoll-Paque™ Plus (GE Healthcare, Freiburg, Germany), were washed in phosphate-buffered saline (PBS)/0.5% bovine serum albumin (BSA) (Sigma-Aldrich, Taufkirchen, Germany), and stained immediately with fluorchrome labeled monoclonal antibodies to a panel of different surface antigens to discriminate PDC, B and T cell subsets as shown in Additional file 1. All samples were analyzed within six hours after acquisition to ensure viability of all cell subsets. To exclude dead cells 4,6-diamidino-2-phenylindole (DAPI, final concentration 220 nM) (Invitrogen, Darmstadt, Germany) was used. A FACS Canto-II and FACS Diva Software (Becton Dickinson, Heidelberg, Germany) were used for a 12-parameter (8-color) flow cytometric analysis. One million events were recorded for B cell or dendritic cell analysis, and 100,000 events for T cell analysis. Results were analyzed using FlowJo Software (Treestar). Lymphocyte counts were recorded and absolute numbers were calculated using the frequencies of PDC, T and B cells based on the lymphocyte gate and the numbers of lymphocytes counted simultaneously in the central laboratory.

### Statistical analysis

We performed an exploratory analysis of a set of serological and cellular parameters obtained from patients receiving MMF and CYC as induction therapy in severe lupus flares. No adjustment for multiple tests was performed.

A cross-sectional analysis was performed and results were confirmed and supplemented by data obtained by a follow-up analysis. Changes of serological and cellular parameters were recorded and compared between different treatment groups.

Data were analyzed using GraphPad Prism 6 (GraphPad Software, Inc.). *P*-values <0.05 were considered to be statistically significant. Since most data did not show a normal distribution, median values with range were applied with few exceptions. The Mann–Whitney test and the Fisher’s exact test were used to compare parameters of two independent unrelated patient groups, and the Wilcoxon’s signed rank test for the follow-up analysis. The Kruskal-Wallis test and the Dunn’s Multiple Comparison test were used if more than two patient groups were compared. Spearman’s rank correlation test was used for the correlation analysis.

## Results

### Cross-sectional analysis

Major impact of CYC or MMF was seen on B cells and B cell subsets. Counts of plasmablasts and plasma cells were significantly lower in patients receiving MMF compared to patients receiving CYC (*P* <0.0001 for both, plasmablasts and plasma cells) and compared to controls (*P* <0.001 for plasmablasts only). Consistent with these findings, serum levels of free light chains, IgG, IgA and IgM were lower in MMF treated patients compared to patients receiving CYC or to controls, but significant differences could only be detected with regard to free kappa light chains (*P* <0.01 compared to CYC and *P* <0.05 compared to controls), free lambda light chains (*P* <0.05 compared to controls), IgA (*P* <0.01 compared to CYC and *P* <0.05 compared to controls), and IgG (*P* <0.05 compared to controls). In contrast, treatment with CYC was associated with significantly lower counts of naïve B cells compared to MMF (*P* <0.05) and with significantly lower counts of total B lymphocytes (*P* <0.05), of pre-switched memory cells (*P* <0.01), and of naïve B cells (*P* <0.0001) compared to controls. We did not observe any significant differences in counts of leukocytes or total lymphocytes in the peripheral blood of patients treated with CYC or MMF compared to controls, nor did we notice any significant differences in T lymphocyte subsets or in counts of circulating PDC. Serological parameters as well as cell subset data are shown in Table 3.

**Table 3:** **Median (range) of serological parameters and cell subsets of patients treated with MMF or CYC compared to controls**

**Madian (range) serological parameters and cell subsets**	**MMF**	**CYC**	**Controls**
	**(number = 25)**	**(number = 20)**	**(number = 22)**
anti-dsDNA (U/ml)	38 (0 to 927)	38 (0 to 7536)	28 (0 to 963)
C3c (g/L)	0.8 (0.4 to 1.1)	0.8 (0.5 to 1.5)	0.8 (0.2 to 1.4)
FLC_kappa_ (mg/L)	19.0 (9.6 to 52.0)^aab^	34.9 (1.6 to 246.0)^aa^	26.7 (9.2 to 148.0)^b^
FLC_lambda_ (mg/L)	21.8 (10.0 to 47.8)^b^	30.2 (4.1 to 153.0)	32.8 (12.9 to 99.3)^b^
IgG (g/L	10.5 (4.9 to 16.3)^b^	11.8 (3.8 to 28.8)	13.4 (5.9 to 24.2)^b^
IgA (g/L)	2.1 (0.2 to 5.7)^aab^	3.3 (1.1 to 6.7)^aa^	2.8 (0.9 to 7.4)^b^
IgM (g/L)	0.9 (0.3 to 5.5)	1.1 (0.3 to 2.6)	1.1 (0.3 to 7.4)
lymphocytes (/μl)	800 (220 to 2160)	790 (230 to 1910)	890 (190 to 2310)
leukocytes (/μl)	6010 (2640 to 11450)	5835 (2400 to 14008)	6195 (2160 to 10700)
platelets (× 10^3^/μl)	259 (170 to 451)^bb^	238 (68 to 355)	211 (47 to 325)^bb^
**CD19** ^**+**^ **B lymphocytes** (/μl)	55.6 (2.8 to 365.0)	31.2 (4.8 to 206.1)^c^	103.0 (14.7 to 277.4)^c^
- CD27^++^CD38^++^ (/μl)	0.8 (0.0 to 7.4)^aaaabb^	7.3 (0.1 to 90.9)^aaaa^	2.5 (0.7 to 61.4)^bb^
- HLADR^high^CD27^++^CD38^++^ (/μl)	0.4 (0.0 to 4.3)^aaaabbb^	4.3 (0.1 to 58.6)^aaaa^	1.8 (0.5 to 55.1)^bbb^
- HLADR^low^CD27^++^CD38^++^ (/μl)	0.3 (0.0 to 3.2)^aaaa^	2.1 (0.0 to 32.3)^aaaa^	1.0 (0.2 to 6.3)
- CD27^+^IgD^−^ (/μl)	4.0 (1.1 to 168.1)	6.2 (0.9 to 47.2)	10.2 (3.8 to 52.6)
- CD27^+^IgD^+^ (/μl)	0.9 (0.1 to 9.0)	0.8 (0.2 to 4.1)^cc^	2.2 (0.4 to 7.7)^cc^
- CD27^−^IgD^+^CD38^+^ (/μl)	26.2 (0.2 to 232.9)^a^	3.2 (0.0 to 106.4)^acccc^	50.7 (2.5 to 118.3)^cccc^
- CD27^−^IgD^−^ (/μl)	10.0 (0.9 to 28.8)	7.0 (1.0 to 38.5)	14.2 (1.7 to 108.5)
- CD27^−^IgD^+^CD38^++^ (/μl)	2.3 (0.0 to 59.1)	2.4 (0.1 to 82.5)	4.9 (0.2 to 48.6)
**CD3** ^**+**^ **T lymphocytes** (/μl)	463.4 (79.9 to 1562.0)	469.1 (120.5 to 1263.0)	616.0 (92.7 to 1675.0)
CD4^+^ (/μl)	317.2 (72.7 to 1142.0)	254.9 (88.6 to 858.2)	445.7 (46.6 to 1307.0)
- CD44^+^CD62L^−^ (/μl)	23.4 (2.2 to 178.6)	26.5 (5.4 to 126.8)	28.5 (5.2 to 334.5)
- CD45RA^−^CD45RO^+^ (/μl)	105.5 (15.4 to 514.9)	120.2 (24.8 to 506.7)	148.2 (22.9 to 754.8)
- CD45RA^+^CD45RO^−^ (/μl)	144.5 (27.7 to 511.3)	119.6 (19.9 to 534.7)	114.9 (19.0 to 633.9)
CD8^+^ (/μl)	112.6 (2.0 to 538.0)	140.8 (17.2 to 334.6)	142.6 (20.9 to 618.0)
- CD44^+^CD62L^−^ (/μl)	21.5 (0.3 to 363.8)	33.4 (4.9 to 227.8)	15.3 (2.8 to 541.4)
- CD45RA^−^CD45RO^+^ (/μl)	16.1 (0.3 to 332.2)	31.5 (5.3 to 142.9)	24.6 (4.7 to 357.8)
- CD45RA^+^CD45RO^−^ (/μl)	77.1 (1.4 to 485.8)	80.6 (10.5 to 250.1)	86.1 (4.9 to 370.5)
CD4^−^CD8^−^ (/μl)	28.0 (2.2 to 97.2)	23.1 (6.2 to 98.4)	22.1 (2.9 to 174.2)
- CD44^+^CD62L^−^ (/μl)	5.7 (0.1 to 71.6)	6.8 (1.5 to 38.3)	6.3 (1.0 to 166.0)
- CD45RA^−^CD45RO^+^ (/μl)	9.2 (0.1 to 43.0)	8.5 (0.5 to 55.8)	10.9 (0.7 to 68.6)
- CD45RA^+^CD45RO^−^ (/μl)	17.9 (0.7 to 70.2)	12.7 (5.0 to 44.6)	13.3 (1.7 to 160.2)
**CD123** ^**+**^ **CD11c** ^**−**^ **HLA-DR** ^**high**^ **PDCs** (/μl)	1.8 (0.4 to 8.5)	2.0 (0.4 to 6.2)	0.8 (0.1 to 20.6)

Statistically significant differences (Dunn’s multiple comparisons test) were observed between patients receiving MMF and CYC (^a^) or controls (^b^) and patients receiving CYC versus controls (^c^) one (*P* <0.05), two (*P* <0.01), three (*P* <0.001), four (P <0.0001) symbols ^abc^. C3c: complement factor C3c; CD4^+^ T cells; CD4^−^CD8^−^: double negative T cells; CD8^+^ T cells; CD27^++^CD38^++^: plasmablasts and plasma cells; CD27^+^IgD^+^: pre-switched memory B cells; CD27^+^IgD^−^: post-switched memory B cells; CD27^−^IgD^+^CD38^+^: naïve B cells; CD27^−^IgD^+^CD38^++^: transitional B cells; CD27^−^IgD^−^: double negative B cells; CD44^+^CD62L^−^: effector T cells; CD45RA^+^CD45RO^−^: naïve T cells; CD45RA^−^CD45RO^+^: memory T cells; CD123^+^CD11c^−^HLA-DR^high^PDCs: plasmacytoid dendritic cells; controls: patients with SLE not receiving MMF or CYC; CYC: cyclophosphamide; FLC: free light chains; HLADR^high^CD27^++^CD38^++^: plasmablasts; HLADR^low^CD27^++^CD38^++^: plasma cells; Ig: immunoglobulin; MMF: mycophenolate mofetil; SLE, systemic lupus erythematosus.

### Follow-up analysis of patients on induction therapy

Although we observed obvious differences with regard to lymphocyte subsets and PDC in patients receiving induction therapy with MMF or CYC, both patient groups showed a comparably good response to treatment. SLEDAI-2 k decreased in both treatment groups (MMF: 12 (6 to 20) versus 9 (0 to 16), (*P* = 0.0051), and CYC: 14 (2 to 30) versus 12 (0 to 18), (*P* = 0.0019).

**Figure 1:**
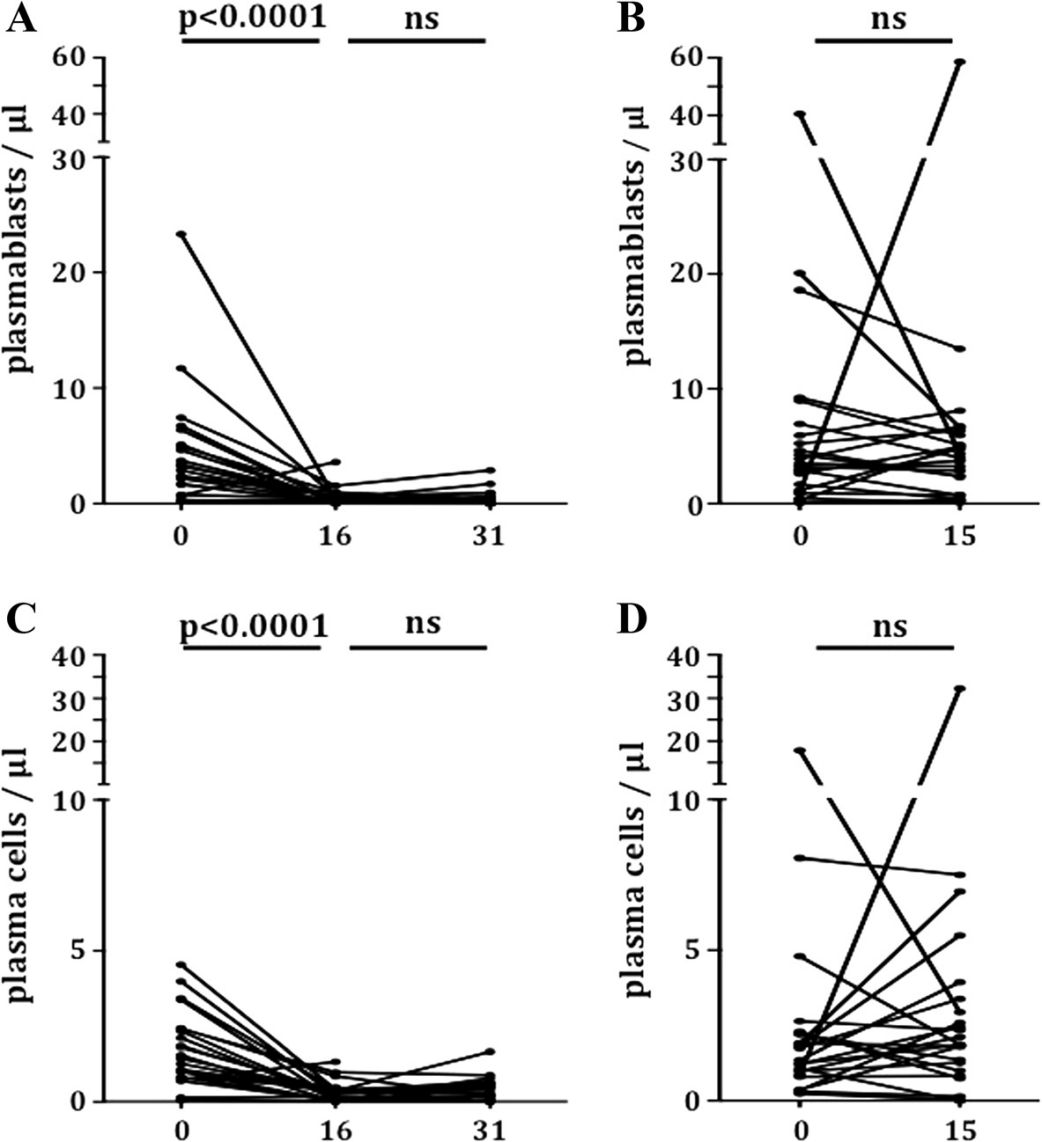
Influence of an induction therapy with mycophenolate mofetil (MMF) or cyclophosphamide (CYC) on plasmablast and plasma cell counts. CD27^++^CD38^++^HLADR^high^ plasmablast **(A)** and CD27^++^CD38^++^HLADR^low^ plasma cell counts **(C)** prior to and approximately 16 and 31 weeks after start of induction therapy with MMF. CD27^++^CD38^++^HLADR^high^ plasmablast **(B)** and CD27^++^CD38^++^HLADR^low^ plasma cell counts **(D)** prior to and approximately 15 weeks after start of induction therapy with CYC. Statistical analyses were performed using the Wilcoxon’s matched pairs signed rank test and *P*-values <0.05 were considered significant.

**Figure 2:**
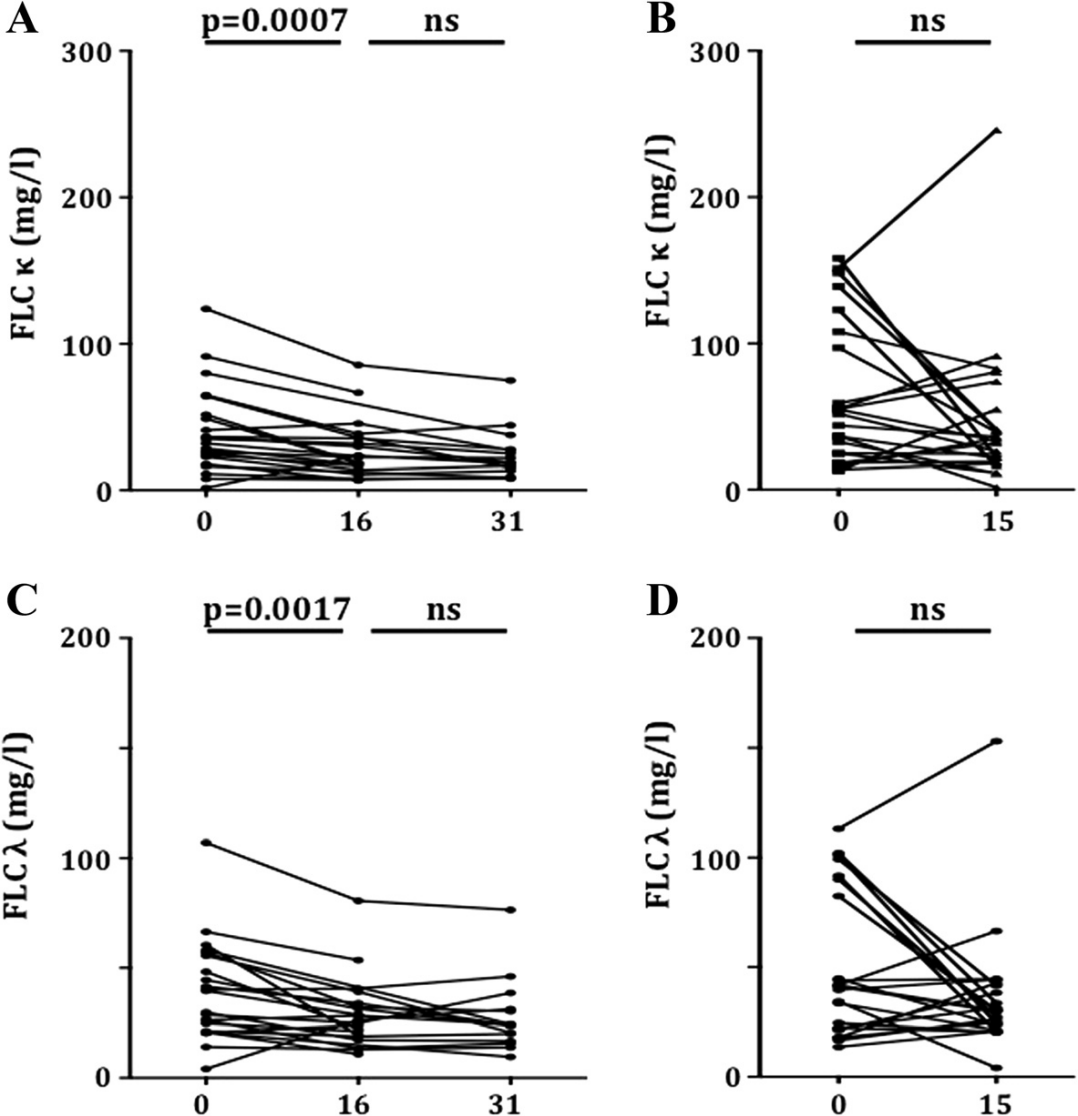
Influence of induction therapy with mycophenolate mofetil (MMF) and cyclophosphamide (CYC) on free light chain (FLC) levels. Levels of free kappa light chains (FLC_kappa_) **(A)** and free lambda light chains (FLC_lambda_) **(C)** prior to and approximately 16 and 31 weeks after start of induction therapy with MMF. Levels of free kappa light chains (FLC_kappa_) **(B)** and free lambda light chains (FLC_lambda_) **(D)** prior to and approximately 15 weeks after start of induction therapy with CYC. Statistical analyses were performed using the Wilcoxon’s matched pairs signed rank test and *P*-values <0.05 were considered significant.

In line with the cross-sectional data, CYC as well as MMF therapy showed a predominant impact on B cell subsets and plasma cells. However, we observed a completely different distribution of B cell subsets comparing both immunosuppressants. In detail, patients receiving MMF showed a fast depletion of plasmablasts (3.2 (0.2 to 23.3) versus 0.3 (0.1 to 3.6)/μl; *P* <0.0001) and of plasma cells (1.4 (0.1 to 4.5) versus 0.3 (0.0 to 1.3)/μl; *P* <0.0001; Figure 1). In the further course of treatment with MMF, both cell subsets remained at a low level (plasmablasts 0.4 (0.0 to 2.8)/μl and plasma cells 0.3 (0.0 to 1.7)/μl). Consistent with these findings levels of free kappa (33.4 (1.6 to 124.0) versus 22.9 (6.7 to 85.5) mg/L) and free lambda (34.7 (4.1 to 107.0) versus 25.7 (10.8 to 80.6) mg/L) light chains declined significantly (*P* = 0.0007 and *P* = 0.0017, respectively), and remained at a low level in the further course of treatment (FLC kappa 19.0 (8.0 to 74.9) mg/L, and FLC lambda 24.0 (9.6 to 76.4) mg/L), whereas no significant decline of free light chain levels was observed in CYC treated patients (Figure 2). In addition, neither counts of plasmablasts nor counts of plasma cells changed significantly after approximately 15 weeks of therapy with CYC (Figure 1).

Levels of IgM decreased significantly in both treatment groups (MMF: 1.1 (0.3 to 4.2) versus 0.9 (0.2 to 4.2) g/L; *P* = 0.0125 and CYC: 1.1 (0.3 to 3.5) versus 1.1 (0.2 to 2.6) g/L; *P* = 0.0214). A statistically significant decline of IgG (12.9 (5.6 to 47.0) versus 10.8 (3.8 to 28.8) g/L; *P* = 0.0250) was only seen in CYC treated patients, whereas MMF treatment was significantly more effective than CYC treatment in reducing the IgA levels (Table 4). Anti-dsDNA antibody titers did not change significantly within approximately three month of induction therapy with CYC or MMF.

**Table 4:** **Direct comparison of MMF and CYC associated changes of cellular and serological parameters during induction therapy**

**Cellular and serological parameters**	**MMF**	**number**	**CYC**	**number**	**Mann–Whitney-test**
anti-dsDNA (U/ml)	−2 (−5134 to +57)	21	−6 (−2135 to +1004)	18	
C3c (g/L)	+0.1 (−0.1 to +0.4)	23	+0.3 (−0.1 to +0.8)	24	*P* = 0.0036
FLC_kappa_ (mg/L)	−9.8 (−38.5 to +21.9)	20	−13.3 (−135.7 to +95.0)	23	
FLC_lambda_ (mg/L)	−10.5 (−41.2 to +22.3)	20	−4.1 (−76.8 to +40.0)	23	
IgG (g/L)	−0.3 (−3.5 to +4.8)	21	−1.3 (−27.5 to +9.1)	23	
IgA (g/L)	−0.3 (−4.2 to +0.9)	15	+0.1 (−1.1 to +3.7)	23	*P* = 0.0127
IgM (g/L)	−0.3 (−0.7 to +1.5)	15	−0.1 (−1.1 to +0.8)	23	
lymphocytes (/μl)	−10 (−500 to +599)	23	−5 (−2040 to +1070)	24	
leukocytes (/μl)	+70 (−6718 to +4000)	23	+460 (−5220 to +10408)	24	
platelets (× 10^3^/μl)	+30 (−116 to +202)	23	0 (−160 to +202)	24	
**CD19** ^**+**^ **B lymphocytes** (/μl)	−2.0 (−146.6 to +110.5)	23	−7.1 (−760.6 to +165.4)	23	*P* = 0.0400
-CD27^++^CD38^++^ (/μl)	−4.1 (−26.7 to +3.4)	23	−0.4 (−51.2 to +89.1)	23	*P* = 0.0014
-HLADR^high^CD27^++^CD38^++^ (/μl)	−2.7 (−22.9 to +2.8)	23	−0.3 (−36.2 to +57.7)	23	*P* = 0.0210
-HLADR^low^CD27^++^CD38^++^ (/μl)	−1.1 (−4.1 -to + 0.6)	23	0.0 (−14.9 to +31.4)	23	*P* = 0.0007
-CD27^+^IgD^−^ (/μl)	−0.3 (−35.2 to +13.1)	22	−2.1 (−123.0 to +49.5)	23	
-CD27^+^IgD^+^ (/μl)	0.0 (−8.3 to +1.4)	22	−0.2 (−11.9 to +3.8)	23	
-CD27^−^IgD^+^CD38^+^ (/μl)	−0.1 (−79.9 to +97.2)	22	−6.5 (−475.7 to +106.4)	23	*P* = 0.0025
-CD27^−^IgD^−^ (/μl)	+0.7 (−26.3 to +17.2)	22	−2.7 (−132.2 to +22.1)	23	*P* = 0.0098
-CD27^−^IgD^+^CD38^++^ (/μl)	+2.5 (−4.8 to +41.6)	23	−0.5 (−61.2 to +63.1)	23	*P* = 0.0107
**CD3** ^**+**^ **T lymphocytes** (/μl)	−14.0 (−466.0 to +363.6)	22	−27.6 (−1360.0 to +813.4)	23	
CD4^+^ (/μl)	−6.4 (−286.6 to +155.5)	22	−91.2 (−1228.0 to +593.4)	23	
-CD44^+^CD62L^−^ (/μl)	−1.7 (−83.3 to +24.9)	22	−1.0 (−111.2 -to +175.2)	23	
-CD45RA^−^CD45RO^+^ (/μl)	+3.3 (−190.6 to +121.4)	22	−16.2 (−492.8 to +377.7)	23	
-CD45RA^+^CD45RO^−^ (/μl)	+10.7 (−220.2 to +53.6)	22	−54.0 (−985.5 to +365.2)	23	
CD8^+^ (/μl)	+8.6 (−262.7 to +264.6)	22	+5.9 (−272.5 to +197.4)	23	
-CD44^+^CD62L^−^ (/μl)	−3.1 (−205.6 to +168.8)	22	+8.3 (−106.2 to +149.3)	23	
-CD45RA^−^CD45RO^+^ (/μl)	−1.1 (−216.1 to +160.1)	22	+5.9 (−180.4 -to + 136.8)	23	
-CD45RA^+^CD45RO^−^ (/μl)	+10.5 (−146.3 to +103.5)	22	+8.2 (−107.9 to +175.0)	23	
CD4^−^CD8^−^ (/μl)	+1.2 (−32.5 ro +63.3)	22	+3.0 (−39.3 to +60.6)	23	
-CD44^+^CD62L^−^ (/μl)	+0.5 (−13.7 to +22.0)	22	+3.0 (−9.8 to +23.5)	23	
-CD45RA^−^CD45RO^+^ (/μl)	+0.5 (−14.6 to +30.2)	22	+1.4 (−13.8 to +36.0)	23	
-CD45RA^+^CD45RO^−^ (/μl)	+0.6 (−12.4 to +32.4)	22	+1.4 (−34.2 to +22.4)	23	
**CD123** ^**+**^ **CD11c** ^**−**^ **HLA-DR** ^**high**^ **PDCs** (/μl)	+0.6 (−4.3 to +6.3)	21	+1.2 (−3.6 to +5.6)	21	

Median values and range are shown. C3c: complement factor C3c; CD4^+^ T cells; CD4^−^CD8^−^: double negative T cells; CD8^+^ T cells; CD27^++^CD38^++^: plasmablasts and plasma cells; CD27^+^IgD^+^: pre-switched memory B cells; CD27^+^IgD^−^: post-switched memory B cells; CD27^−^IgD^+^CD38^+^: naïve B cells; CD27^−^IgD^+^CD38^++^: transitional B cells; CD27^−^IgD^−^: double negative B cells; CD44^+^CD62L^−^: effector T cells; CD45RA^+^CD45RO^−^: naïve T cells; CD45RA^−^CD45RO^+^: memory T cells; CD123^+^CD11c^−^HLA-DR^high^PDCs: plasmacytoid dendritic cells; CYC: cyclophosphamide; FLC: free light chains; HLADR^high^CD27^++^CD38^++^: plasmablasts; HLADR^low^CD27^++^CD38^++^: plasma cells; Ig: immunoglobulin; MMF: mycophenolate mofetil.

While transitional B cells only increased significantly after MMF therapy was initiated (0.8 (0.1 to 8.7) versus 3.9 (0.1 to 43.3)/μl; *P* = 0.0015), neither CYC nor MMF medication affected pre- or post-switched memory B cells significantly within 15 weeks of therapy. In contrast, CYC, but not MMF, treatment was accompanied by a significant decline in counts of total B lymphocytes (66.6 (13.3 to 802.2) versus 31.2 (3.0 to 206.1)/μl; *P* = 0.0327), of naïve B cells (16.5 (0.0 to 481.3) versus 3.2 (0.0 to 106.4)/μl; *P* = 0.0059), and of double negative (CD27^−^IgD^−^) B cells (14.5 (2.9 to 139.6) versus 5.0 (0.7 to 38.5)/μl; *P* = 0.0169). Furthermore, we observed a significant increase of circulating PDC (1.0 (0.0 to 4.3) versus 1.9 (0.2 to 6.2)/μl; *P* = 0.0158) exclusively in the peripheral blood of patients undergoing treatment with CYC. Another observation only made in patients treated with CYC was a significant increase of circulating CD8^+^CD44^+^CD62L^−^ effector T cells (19.9 (2.2 to 157.6) versus 35.5 (4.9 to 227.8)/μl; *P* = 0.0384). Neither MMF nor CYC therapy affected other T lymphocyte subsets in the peripheral blood significantly. Detailed information is shown in Additional files 2 and 3 and a direct comparison of the differences observed between MMF and CYC treatment is shown in Table 4.

### Correlation of free light chain levels, plasmablasts and disease activity

In order to investigate if cellular or serological parameters correlate with each other or with disease activity, we analyzed additional data obtained by monitoring treatment of our lupus cohort. The results of Spearman’s rank correlation test performed with data obtained from 186 patients are shown in Table 5. The disease activity (SLEDAI-2k) correlated significantly with HLADR^high^CD27^++^CD38^++^ plasmablast counts and free light chain levels but not with IgG levels. In addition, HLADR^high^CD27^++^CD38^++^ plasmablast counts correlated significantly with levels of free light chains.

**Table 5:** **Results of a correlation analysis including cellular and serological parameters as well as SLEDAI-2k**

		**SLEDAI-2k**	**HLADR** ^**high**^ **CD27** ^**++**^ **CD38** ^**++**^ **(/μl)**	**FLC**_**kappa**_	**FLC**_**lambda**_	**IgG**	**IgA**
HLADR^high^CD27^++^CD38^++^ (/μl)	p	0.0350					
	r_s_	0.1547					
FLC_kappa_	p	0.0004	0.0002				
	r_s_	0.2591	0.2760				
FLC_lambda_	p	0.0002	0.0003	<0.0001			
	r_s_	0.2726	0.2655	0.8479			
IgG	p	ns	0.0002	<0.0001	<0.0001		
	r_s_		0.2703	0.5105	0.4328		
IgA	p	ns	<0.0001	< 0.0001	< 0.0001	< 0.0001	
	r_s_		0.3306	0.3536	0.3950	0.3027	
IgM	p	ns	ns	0.0423	0.0117	ns	ns
	r_s_			0.1528	0.1890		

Data from 186 patients with SLE are shown. Results were determined by nonparametric Spearman correlation; FLC: free light chains; Ig: immunoglobulin; HLADR^high^CD27^++^CD38^++^: plasmablasts; SLEDAI-2 k: SLE disease activity index; SLE, systemic lupus erythematosus.

## Discussion

The results of the current study indicate a predominant influence of MMF and CYC on B cell subsets. In addition, CYC had an impact on numbers of circulating PDC and CD8^+^CD44^+^CD62L^−^ effector T cells, whereas other T cell subsets were neither affected significantly by treatment with MMF nor by CYC therapy. During the early phase of induction therapy MMF leads to a fast reduction of plasmablasts and plasma cells, whereas CYC has no significant influence on these B cell subsets. The latter has also been observed in mice suggesting a resistance of plasma cells to CYC [21]. Circulating plasmablasts correlated significantly with free light chains in our lupus cohort. Consistent with these findings, free light chains as a surrogate marker for plasma cell activation were only influenced significantly by treatment with MMF but not with CYC. A significant reduction of free light chain concentrations over time is a phenomenon also observed in patients treated with the B cell depleting antibody rituximab [22]. In SLE patients treated with MMF it is probably the consequence of a predominant and fast inhibiting influence of mycophenolic acid on the synthesis of plasmablasts [16,17]. The fast turnover of free light chains with a plasma half-life period of two to four hours for FLC_kappa_ and three to six hours for FLC_lambda_ [23] compared to the long plasma half-life period of about 21, 10 and 6 days for IgG, IgM and IgA, respectively [24] predestines them to be used as a marker of disease activity and early response to therapy [25,26]. In addition, plasmablasts and free light chains might also serve as indicators of upcoming flares but this remains to be investigated in longitudinal studies enrolling patients with stable disease. In this context, Hopper *et al*. reported a considerable time period between the onset of light chain secretion into the urine and the occurrence of inflammation [27].

Plasmablasts as well as lambda and kappa light chains correlated significantly with the SLEDAI-2k in our lupus cohort, whereas no correlation was observed between SLEDAI-2k and serum levels of IgG, IgM and IgA. An association of disease activity and free light chains has already been described in patients with SLE [22,28] or other autoimmune disorders, such as rheumatoid arthritis or Sjögren’s syndrome [26]. Thus, monitoring of plasmablasts and free light chains might be an effective tool to predict response to treatment or flares.

As previously shown in patients receiving CYC for longer periods of time, CYC does finally also affect plasmablast counts [8]. Considering the additional data obtained by the current study this seems to be an indirect and rather slow process probably related to an impact on other cells than memory B cells or plasmablasts.

The opposite influence of MMF and CYC on IgA levels might suggest a distinct mechanism of action of both drugs on different subsets of antibody-forming cells, or might simply be a consequence of a higher turnover rate of IgA+ plasmablasts in patients with SLE. Both IgG and IgA can activate Fc-receptors, and autoantibodies of both isotypes seem to be associated with active disease in SLE or antiphospholipid syndrome [29-32]. IgA secreting plasmablasts make up 44% to 86% of all circulating antibody forming cells in patients with SLE [33], and it has recently been reported that T2 B cells from patients with SLE are unable to reach the gut associated lymphoid tissue (GALT) which lacks IgA secreting cells in SLE. This was suggested to abrogate an important checkpoint of B cell tolerance in SLE [34]. MMF is apparently able to inhibit plasmablast generation directly and quickly and regardless of the character and origin of the plasmablasts. In contrast, CYC influences it comparatively slowly by diminishing precursor cells, such as naïve or transitional B cells.

Although SLE is a disease of enhanced antibody production and is often associated with hypergammaglobulinemia [35], we noticed patients with low Ig levels in both treatment groups as well as in the control group not receiving CYC or MMF. In contrast to prior findings that MMF [36], but not CYC, therapy [37] is associated with severe hypogammaglobulinemia, we observed a significant decline of IgG and IgM after CYC, but only of IgM after MMF was initiated. The decrease of Ig levels after CYC treatment has also been described in patients with anti-neutrophil cytoplasmic antibody (ANCA)- associated vasculitis [38]. The lack of immunoglobulins might be explained by the high proportion of proteinuric patients, as hypogammaglobulinemia is a common finding in patients with nephrotic syndrome [39]. In addition, patients receiving CYC or MMF for induction therapy were treated with a relatively high dose of corticosteroids causing lower IgG levels as well [40]. Altogether, our data suggest that especially patients with proteinuria should be monitored closely with regard to Ig levels and infections no matter if treated with MMF or CYC. Especially a combination with other immunosuppressive agents, such as atacicept [41] or ocrelizumab [42], has been described to be associated with a higher risk for development of severe immunoglobulin deficiency and infections.

Although lymphopenia and alterations of B cell subsets have already been described in SLE [9,10,43], this manuscript contributes to a better understanding of the influence of the two most important immunosuppressants used for induction therapy in patients with organ-threatening disease. Induction therapy with CYC was associated with a preferential depletion of naïve, double negative (CD27^−^IgD^−^) and pre-switched memory B cells, whereas treatment with MMF was not accompanied by a depletion of transitional or naïve B cells. Hence, especially naïve B cells seem to be more susceptible to CYC, a phenomenon also seen in patients with ANCA associated vasculitis receiving CYC for induction therapy [44] and in another SLE cohort [8]. Numbers of transitional B cells increased significantly after MMF was started, while they decreased after CYC was initiated, suggesting that MMF, in contrast to other immunosuppressants, spares transitional B cells. Post-switched memory cells were not influenced by either of these therapies significantly. This is in line with the findings of other previous studies [8,16] and might be related to the fact that memory cells are resting cells or might sit in niches protecting them from depletion [45].

In the context of induction therapy with CYC or MMF we observed a significant increase in the levels of circulating PDC and CD8^+^CD44^+^CD62L^−^ effector T cells only in patients treated with CYC. There is evidence for CD8^+^ effector T cells accumulating in inflamed kidneys and causing glomerular injury in a mouse model of systemic lupus [46] as well as in patients with severe lupus nephritis [47]. The frequency of circulating PDC is known to be low in SLE patients [15]. A migration of PDC into inflamed organs or tissues (that is, the kidneys of SLE patients with active nephritis) [48,49] or cutaneous lesions [50,51] has been discussed as an explanation of the low frequencies of PDC observed in the peripheral blood of patients with SLE during disease flares. Since we did not observe a significant drop of PDC or CD8^+^CD44^+^CD62L^−^ effector T cells after MMF was introduced, our data suggest that CYC treatment might be able to ameliorate tissue inflammation more efficiently or more rapidly than MMF and might lead to an earlier consecutive reappearance of PDCs or CD8^+^CD44^+^CD62L^−^ effector T cells in the peripheral blood. These observations suggest that both drugs, used in similar occasions, such as lupus nephritis, with comparable results act in a quite different manner. In addition, these findings suggest that one or the other drug might be more or less effective in certain patients depending on individual features, such as their level of B cell activation or degree of tissue infiltration by activated T cells. To customize therapy in patients with SLE, biomarkers allowing the identification of the driving force of inflammation in individual patients are needed. Free light chains or plasmablasts might be used to identify candidates who might profit from MMF treatment.

The current study has limitations because of its observational and retrospective character. Corticosteroids and disease activity are known to affect circulating immune cell subsets, as for instance DCs or lymphocytes. Besides activity and organ involvement, treatment with corticosteroids has been shown to diminish levels of circulating PDC in SLE [52]. Therefore, we compared treatment groups with a similar median corticosteroid dose.

Focused on corticosteroid dose and disease activity as major factors influencing lymphocyte and DC subsets in SLE, other potential confounders, such as organ manifestations, were not considered (Tables 1 and 2). In addition, the local situation concerning the lack of approval of MMF but not CYC for treating SLE and lupus nephritis might explain further differences between the groups receiving MMF or CYC, as for instance a low rate of pretreated patients within the CYC group.

## Conclusions

Regardless of these limitations, our data suggest differences between MMF and CYC with regard to the mechanism of action. MMF, but not CYC, treatment leads to a fast and enduring reduction of surrogate markers for B cell activation, such as circulating plasmablasts, plasma cells, and free light chains. The data might help to pave the way for more customized therapies in SLE and the impact of MMF and CYC on cellular and serological parameters should be considered when biomarker panels for clinical trials are discussed and free light chains or plasmablasts and plasma cells are monitored. In contrast, we did not observe a significant difference between CYC and MMF in inducing low IgG levels during induction therapy over a time frame of three months.

## Additional files

Additional file 1:
**List of antibodies used for flow cytometrical analysis.**
Additional file 2:
**Follow-up data of patients receiving MMF.**
Additional file 3:
**Follow-up data of patients receiving CYC.**

## Abbreviations

ACR: American College of Rheumatology
ANA: antinuclear antibodies
anti ds-DNA: anti double-stranded DNA
AZA: azathioprine
BSA: bovine serum albumin
CD: cluster of differentiation
CYC: cyclophosphamide
DC: dendritic cell
DMARD: disease modifying antirheumatic drug
FACS: fluorescence activated cell sorting
FLC: free light chain
HLADR: human leukocyte antigen-DR
Ig: immunoglobulin
MMF: mycophenolate mofetil
MTX: methotrexate
PBS: phosphate-buffered saline
PDC: plasmacytoid dendritic cells
PMBC: peripheral blood mononuclear cells
SLE: systemic lupus erythematosus
SLEDAI-2k: systemic lupus erythematosus disease activity index 2k
T2: transitional type 2
