# Supplementary material for: Differential effects of cyclophosphamide and mycophenolate mofetil on cellular and serological parameters in patients with systemic lupus erythematosus
Source: Arthritis Res Ther. 2015 Apr 3;17(1):92. doi: 10.1186/s13075-015-0603-8 (PMC4422597; doi:10.1186/s13075-015-0603-8)
Supplement: Additional file 1: — List of antibodies used for flow cytometrical analysis. [file 13075_2015_603_MOESM1_ESM.pdf]

name	clone	format / label	source
CD3	UCHT1	Pacific Blue	BD
CD4	RPA-T4	APC-H7	BD
CD8	RPA-T8	Horizon V500	BD
CD11c	B-ly6	APC	BD
CD14	M5E2	Pacific Blue	BD
CD19	SJ25C1	PE-Cy7	BD
CD20	L27	Horizon V450	BD
CD27	L128	APC	BD
CD38	HB-7	PE	BD
CD44	G44-26	PerCP-Cy5.5	BD
CD45RA	HI100	PE-Cy7	BD
CD45RO	UCHL1	APC	BD
CD56	B159	Horizon V450	BD
CD62L	Dreg56	FITC	BD
CD123	7G3	PerCP-Cy5.5	BD
HLA-DR	TÜ36	Pacific Orange	Inv.
HLA-DR	G46-6	PE-Cy7	BD
IgD	IA6-2	Biotin/ SA-AF680	BD/Inv.

APC = allophycocyanin; BD: Becton Dickinson, USA; FITC= fluorescein isothiocyanate; Inv.: Invitrogen, USA; PE= phycoerythrin; PerCP = peridinin chlorophyll, SA = streptavidin, AF = alexa-fluor
