# Supplementary material for: Differential effects of cyclophosphamide and mycophenolate mofetil on cellular and serological parameters in patients with systemic lupus erythematosus
Source: Arthritis Res Ther. 2015 Apr 3;17(1):92. doi: 10.1186/s13075-015-0603-8 (PMC4422597; doi:10.1186/s13075-015-0603-8)
Supplement: Additional file 2: — Follow-up data of patients receiving MMF. [file 13075_2015_603_MOESM2_ESM.pdf]

Serological and cellular parameters presented as median and range of 23 patients prior to and 16 (10-65) weeks after start of induction therapy with MMF.

	n	prior to MMF	with MMF	Wilcoxon's matched pairs signed rank test
anti-dsDNA (U/ml)	21	31 (4-7536)	26 (6-2402)	
C3c (g/l)	23	0.8 (0.3-1.2)	0.9 (0.4-1.3)	p=0.0005
FLC_{kappa} (mg/l)	20	33.4 (1.6-124.0)	22.9 (6.7-85.5)	p=0.0007
FLC_{lambda} (mg/l)	20	34.7 (4.1-107.0)	25.7 (10.8-80.6)	p=0.0017
IgG (g/l)	21	*11.6* (3.8-22.1)	10.5 (4.7-23.4)	
IgA (g/l)	15	3.2 (0.8-6.0)	2.6 (0.5-5.3)	
IgM (g/l)	15	1.1 (0.3-4.2)	0.9 (0.2-4.2)	p=0.0125
lymphocytes (/µl)	23	550 (100-1670)	650 (190-1430)	
leukocytes (/µl)	23	5480 (1240-14008)	5400 (3000-9070)	
platelets (x 10^3/µl)	23	255 (107-355)	258 (188-453)	
**CD19^{+} B lymphocytes** (/µl)	23	22.9 (2.0-214.3)	37.0 (5.5-138.4)	
- CD27^{++}CD38^{++} (/µl)	23	4.4 (0.3-27.3)	0.6 (0.0-4.9)	p<0.0001
- HLADR^{high}CD27^{++}CD38^{++} (/µl)	23	3.2 (0.2-23.3)	0.3 (0.1-3.6)	p<0.0001
- HLADR^{low}CD27^{++}CD38^{++} (/µl)	23	1.4 (0.1-4.5)	0.3 (0.0-1.3)	p<0.0001
- CD27^{+}IgD^{-} (/µl)	22	6.2 (0.9-47.2)	6.4 (1.5-26.8)	
- CD27^{+}IgD^{+} (/µl)	22	0.7 (0.2-10.6)	0.6 (0.1-5.8)	
- CD27^{-}IgD^{+}CD38^{+} (/µl)	22	5.9 (0.0-117.0)	7.5 (0.0-97.8)	
- CD27^{-}IgD^{-} (/µl)	22	5.0 (0.7-42.9)	7.9 (1.7-35.8)	
- CD27^{-}IgD^{+}CD38^{++} (/µl)	23	0.8 (0.1-8.7)	3.9 (0.1-43.3)	p=0.0015
**CD3^{+} T lymphocytes** (/µl)	22	401.7 (120.5-1221.0)	407.4 (85.5-1185.0)	
CD4^{+} (/µl)	22	230.4 (88.6-858.2)	265.3 (68.3-754.0)	
- CD44^{+}CD62L^{-} (/µl)	22	28.9 (2.1-203.7)	24.7 (6.0-120.4)	
- CD45RA^{-}CD45RO^{+} (/µl)	22	99.0 (29.0-490.8)	95.6 (24.5-300.3)	
- CD45RA^{+}CD45RO^{-} (/µl)	22	108.7 (19.9-534.7)	118.5 (28.6-492.3)	
CD8^{+} (/µl)	22	119.1 (17.2-380.9)	117.8 (9.4-368.7)	
- CD44^{+}CD62L^{-} (/µl)	22	15.2 (3.0-319.6)	18.4 (1.1-188.4)	
- CD45RA^{-}CD45RO^{+} (/µl)	22	25.6 (4.7-259.7)	24.0 (2.2-178.7)	
- CD45RA^{+}CD45RO^{-} (/µl)	22	63.9 (9.9-349.3)	90.5 (7.0-324.8)	
CD4^{-}CD8^{-} (/µl)	22	16.5 (6.4-85.9)	16.0 (3.3-87.4)	
- CD44^{+}CD62L^{-} (/µl)	22	4.9 (0.3-33.0)	4.9 (0.8-47.3)	
- CD45RA^{-}CD45RO^{+} (/µl)	22	4.7 (1.0-48.2)	6.6 (0.5-47.6)	
- CD45RA^{+}CD45RO^{-} (/µl)	22	9.9 (2.9-26.5)	9.5 (2.0-39.5)	
**CD123^{+}CD11c^{-}HLADR^{high}PDCs** (/µl)	21	1.5 (0.3-5.2)	1.9 (0.4-7.0)	

CD4^{+} T cells; CD4^{-}CD8^{-}: double negative T cells; CD8^{+} cytotoxic T cells; CD27^{++}CD38^{++}: plasmablasts and plasma cells; CD27^{+}IgD^{+}: pre-switched memory B cells; CD27^{+}IgD^{-}: post-switched memory B cells; CD27^{-}IgD^{+}CD38^{+}: naïve B cells; CD27^{-}IgD^{+}CD38^{++}: transitional B cells; CD27^{-}IgD^{-}: double negative B cells; CD44^{+}CD62L^{-}: effector T cells; CD45RA^{+}CD45RO^{-}: naïve T cells; CD45RA^{-}CD45RO^{+}: memory T cells; CD123^{+}CD11c^{-}HLADR^{high}PDCs: plasmacytoid dendritic cells; FLC: free light chains; HLADR^{high}CD27^{++}CD38^{++}: plasmablasts; HLADR^{low}CD27^{++}CD38^{++}: plasma cells; HLADR$^{low+high}$CD27^{++}CD38^{++}: plasmablasts and plasma cells; C3c: complement factor C3c; Ig: immunoglobulin; MMF: mycophenolate mofetil.
