# Supplementary material for: Differential effects of cyclophosphamide and mycophenolate mofetil on cellular and serological parameters in patients with systemic lupus erythematosus
Source: Arthritis Res Ther. 2015 Apr 3;17(1):92. doi: 10.1186/s13075-015-0603-8 (PMC4422597; doi:10.1186/s13075-015-0603-8)
Supplement: Additional file 3: — Follow-up data of patients receiving CYC. [file 13075_2015_603_MOESM3_ESM.pdf]

Serological and cellular parameters presented as median and range of 24 patients prior to and 15 (10-39) weeks after start of induction therapy with CYC.

	n	prior to CYC	with CYC	Wilcoxon's matched pairs signed rank test
anti-dsDNA (U/ml)	18	176 (5-2147)	38 (4-1250)	
C3c (g/l)	24	0.5 (0.1-1.4)	0.8 (0.5-1.5)	$p<0.0001$
FLC$_{kappa}$ (mg/l)	23	51.7 (12.8-158.0)	33.2 (1.6-246.0)	
FLC$_{lambda}$ (mg/l)	23	41.0 (13.6-113.0)	29.8 (4.1-153.0)	
IgG (g/l)	23	12.9 (5.6-47.0)	10.8 (3.8-28.8)	$p=0.0250$
IgA (g/l)	23	2.5 (0.7-6.4)	3.3 (0.8-6.7)	
IgM (g/l)	23	1.1 (0.3-3.5)	1.1 (0.2-2.6)	$p=0.0214$
lymphocytes (/µl)	24	695 (220-2370)	750 (100-1910)	
leukocytes (/µl)	24	4810 (1620-9980)	5835 (2400-14008)	
platelets (x 10^{3}/µl)	24	224 (67-365)	238 (68-355)	
**CD19^{+} B lymphocytes** (/µl)	23	66.6 (13.3-802.2)	31.2 (3.0-206.1)	$p=0.0327$
- CD27^{++}CD38^{++} (/µl)	23	5.4 (0.4-58.4)	7.3 (0.1-90.9)	
- HLADR^{high}CD27^{++}CD38^{++} (/µl)	23	3.5 (0.1-40.6)	4.3 (0.1-58.6)	
- HLADR^{low}CD27^{++}CD38^{++} (/µl)	23	1.4 (0.2-17.8)	1.9 (0.0-32.3)	
- CD27^{+}IgD^{-} (/µl)	23	11.0 (2.4-137.2)	6.5 (0.9-73.1)	
- CD27^{+}IgD^{+} (/µl)	23	1.3 (0.2-12.7)	0.8 (0.2-8.8)	
- CD27^{-}IgD^{+}CD38^{+} (/µl)	23	16.5 (0.0-481.3)	3.2 (0.0-106.4)	$p=0.0059$
- CD27^{-}IgD^{-} (/µl)	23	14.5 (2.9-139.6)	5.0 (0.7-38.5)	$p=0.0169$
- CD27^{-}IgD^{+}CD38^{++} (/µl)	23	3.3 (0.1-61.7)	1.7 (0.1-82.5)	
**CD3^{+} T lymphocytes** (/µl)	23	489.8 (178.9-1626.0)	472.0 (120.5-1263.0)	
CD4^{+} (/µl)	23	361.0 (111.1-1397.0)	252.2 (88.6-858.2)	
- CD44^{+}CD62L^{-} (/µl)	23	28.5 (5.5-237.9)	28.0 (5.4-203.7)	
- CD45RA^{-}CD45RO^{+} (/µl)	23	121.0 (37.6-602.7)	125.2 (24.8-506.7)	
- CD45RA^{+}CD45RO^{-} (/µl)	23	169.5 (35.6-1124.0)	110.1 (19.9-534.7)	
CD8^{+} (/µl)	23	139.7 (26.1-331.6)	152.8 (17.2-334.6)	
- CD44^{+}CD62L^{-} (/µl)	23	19.9 (2.2-157.6)	35.5 (4.9-227.8)	$p=0.0384$
- CD45RA^{-}CD45RO^{+} (/µl)	23	26.9 (4.2-199.0)	32.2 (5.3-142.9)	
- CD45RA^{+}CD45RO^{-} (/µl)	23	71.3 (21.5-274.1)	80.8 (10.5-250.1)	
CD4^{-}CD8^{-} (/µl)	23	28.2 (4.7-63.6)	24.3 (6.2-98.4)	
- CD44^{+}CD62L^{-} (/µl)	23	6.0 (0.9-18.1)	7.1 (1.5-38.3)	
- CD45RA^{-}CD45RO^{+} (/µl)	23	9.4 (1.7-27.0)	9.3 (0.5-55.8)	
- CD45RA^{+}CD45RO^{-} (/µl)	23	17.3 (3.0-51.3)	13.3 (5.0-44.6)	
**CD123^{+}CD11c^{-}HLADR^{high}PDCs** (/µl)	21	1.0 (0.0-4.3)	1.9 (0.2-6.2)	$p=0.0158$

CD4^{+} T cells; CD4^{-}CD8^{-}: double negative T cells; CD8^{+} cytotoxic T cells; CD27^{++}CD38^{++}: plasmablasts and plasma cells; CD27^{+}IgD^{+}: pre-switched memory B cells; CD27^{+}IgD^{-}: post-switched memory B cells; CD27^{-}IgD^{+}CD38^{+}: naïve B cells; CD27^{-}IgD^{+}CD38^{++}: transitional B cells; CD27^{-}IgD^{-}: double negative B cells; CD44^{+}CD62L^{-}: effector T cells; CD45RA^{+}CD45RO^{-}: naïve T cells; CD45RA^{-}CD45RO^{+}: memory T cells; CD123^{+}CD11c^{-}HLADR^{high}PDCs: plasmacytoid dendritic cells; FLC: free light chains; HLADR^{high}CD27^{++}CD38^{++}: plasmablasts; HLADR^{low}CD27^{++}CD38^{++}: plasma cells; HLADR$^{low+high}$CD27^{++}CD38^{++}: plasmablasts and plasma cells; C3c: complement factor C3c; Ig: immunoglobulin; MMF: mycophenolate mofetil.
